# Impaired Mitophagy Plays a Role in Denervation of Neuromuscular Junctions in ALS Mice

**DOI:** 10.3389/fnins.2017.00473

**Published:** 2017-08-25

**Authors:** Robert S. Rogers, Sudheer Tungtur, Tomohiro Tanaka, Lisa L. Nadeau, Yomna Badawi, Hua Wang, Hong-Min Ni, Wen-Xing Ding, Hiroshi Nishimune

**Affiliations:** ^1^Department of Anatomy and Cell Biology, University of Kansas School of Medicine Kansas City, KS, United States; ^2^Department of Pharmacology, Toxicology and Therapeutics, University of Kansas School of Medicine Kansas City, KS, United States

**Keywords:** ALS, autophagy, denervation, mitophagy, NMJ, p62/SQSTM1, SOD1^G93A^ mice

## Abstract

Motor neurons in amyotrophic lateral sclerosis (ALS) patients and animal models show degeneration from the nerve terminal, known as dying-back neuropathy. To investigate the mechanism underlying this neuropathy, we analyzed the neuromuscular junctions (NMJs) and motor neuron cell bodies in SOD1^G93A^ mice using electron microscopy. NMJs of SOD1^G93A^ mice exhibited significantly higher numbers of autophagosomes and degenerated mitochondria compared to wild-type controls. Mitophagosomes were identified in the NMJ presynaptic terminals of wild-type mice and SOD1^G93A^ mice. However, the number of mitophagosomes did not increase significantly in SOD1^G93A^ NMJs indicating a defect in mitophagy, the autophagic process to degrade mitochondria. Consistent with this, proteins essential for mitophagy, p62/SQSTM1, Bnip3, Pink1, and Parkin were down-regulated in motor neurons in SOD1^G93A^ mice. Importantly, SQSTM1 is one of the genes mutated in familial ALS patients. We evaluated the effect of impaired mitophagy on motor neurons by analyzing the double knockout mice of Pink1 and Parkin, two genes responsible for sensing depolarized mitochondria and delivering degenerated mitochondria to mitophagosomes. The double knockout mice exhibited NMJ degeneration, including axon swelling and NMJ fragmentation at 4 months of age. These phenotypes were rarely observed in wild-type control mice of the same age. The protein level of ATP synthase β subunit increased in the NMJ presynaptic terminals, suggesting the accumulation of mitochondria at NMJs of the double knockout mice. Importantly, NMJ denervation was observed in the double knockout mice. These data suggest that the reduced mitophagy function in motor neurons of SOD1^G93A^ mice is one of the mechanisms causing degeneration of ALS NMJs.

## Introduction

Amyotrophic lateral sclerosis (ALS) is a common and fatal motor neuron disease, but the etiology has not been elucidated fully (Bordet et al., 2001; Acsadi et al., 2002; Kaspar et al., 2003; Azzouz et al., 2004; Gould et al., 2006; Sorenson et al., 2008; Howe et al., 2009; Reyes et al., 2010; Jeong et al., 2011). In familial ALS patients, causal or risk-increasing roles in the pathogenesis of ALS have been reported for mutations in autophagy- and mitophagy-related genes, including ALS2/Alsin, charged multivesicular protein 2B (CHMP2B), C9orf72, dynactin 1 (DCTN1), Optineurin, p62/SQSTM1 (sequestosome 1), Ubiquilin 2, and Valosin-containing protein (VCP) (Yang et al., 2001; Munch et al., 2004; Parkinson et al., 2006; Johnson et al., 2010; Maruyama et al., 2010; DeJesus-Hernandez et al., 2011; Deng et al., 2011; Fecto et al., 2011; Rubino et al., 2012; Williams et al., 2012; Farg et al., 2014). Autophagy is a selective process by which proteins and organelles are enveloped by a double membrane structure, the autophagosome, for delivery to lysosomes for degradation (Klionsky et al., 2016). The autophagy markers microtubule-associated protein 1 light chain 3 (LC3) and p62/SQSTM1 were detected in aggregated structures in spinal cord motor neurons of sporadic ALS patients suggesting a modulation of autophagy-related proteins in ALS (Sasaki, 2011). Interestingly, mice with nervous system-restricted knockout of the autophagy-essential genes *Atg5* or *Atg7* exhibited motor impairment, including impaired coordination and balance, reduced grip strength by 3–4 weeks of age, and axon degeneration (Hara et al., 2006; Komatsu et al., 2006, 2007). Nervous system-restricted knockout of the autophagy genes *Atg5* or *Atg9a* exhibited axon degeneration of central nervous system neurons (Nishiyama et al., 2007; Yamaguchi et al., 2017). Together, these observations suggest that autophagy dysfunction contributes to the etiology of ALS (Otomo et al., 2012; Ruffoli et al., 2015; Edens et al., 2016).

In SOD1^G93A^ mice, an ALS animal model, a defect of autophagosome fusion with lysosomes has been reported in the motor neuron cell body (Xie et al., 2015). Furthermore, electron microscopy analyses of motor neurons in SOD1^G93A^ mice and ALS patients have revealed autophagic vacuoles possibly arising from altered mitochondria (Hart et al., 1977; Wong et al., 1995). Increasing evidence suggests that defective mitochondrial function and impaired autophagy play roles in ALS etiology (Edens et al., 2016). Degenerated mitochondria are removed by a specific autophagic mechanism called mitophagy, which targets degenerated mitochondria (Youle and Narendra, 2011; Ding and Yin, 2012). Mitophagy is mediated by the following two major pathways. PTEN-induced putative kinase 1 (PINK1, *pink1*) is normally degraded rapidly, but it is stabilized at the surface of depolarized mitochondria, where it recruits and activates Parkin (a component of E3 ubiquitin ligase, *park2*; Matsuda et al., 2010; Narendra et al., 2010). Activated Parkin ubiquitinates outer mitochondrial membrane proteins including mitofusin 1/2 and voltage-dependent anion-selective channel protein 1 (VDAC1; Geisler et al., 2010). p62/SQSTM1 has a ubiquitin binding domain at the C-terminus that recognizes ubiquitinated mitochondrial proteins, accumulates to depolarized mitochondria, and recruits LC3 to mitochondria to induce mitophagy (Geisler et al., 2010; Okatsu et al., 2010). Phagophores are recruited by LC3 to form autophagosomes, and LC3 becomes lipidated to form LC3-II, which is a marker for autophagy levels (Kabeya et al., 2000; Klionsky et al., 2016). In a separate pathway, Nix and BCL2/adenovirus E18 19 kDa protein-interacting protein 3 (Bnip3) are also responsible for recruiting LC3 to the mitochondria to initiate mitophagy (Ding et al., 2010; Hanna et al., 2012). However, the functional level of mitophagy in ALS motor neurons is not well known.

Does mitophagy play a role in ALS etiology, and if so, where in motor neurons would mitophagy cause degeneration? Motor neuron degeneration is preceded by denervation of neuromuscular junctions (NMJs) in ALS patients and animal models, producing what is known as a dying-back neuropathy (Kennel et al., 1996; Siklos et al., 1996; Frey et al., 2000; Fischer et al., 2004; Dadon-Nachum et al., 2011). Furthermore, degenerated mitochondria have been detected at motor neuron presynaptic terminals of SOD1^G93A^ mice (Gould et al., 2006), suggesting that mitochondrial degeneration at the NMJs plays a role in this dying-back neuropathy. Interestingly, NMJ denervation cannot be prevented by blocking motor neuron apoptosis in ALS mice, suggesting that a mechanism other than apoptosis is responsible for the degeneration of axons and NMJs (Gould et al., 2006; Reyes et al., 2010). Therefore, degenerative changes in ALS motor neurons occur at the NMJ. However, the roles of autophagy and mitophagy at the NMJ have not been explored extensively. In this study, we performed ultrastructural analysis to investigate autophagosomes and mitophagosomes at NMJs. We also tested the role of mitophagy in the maintenance of NMJs by depleting both Pink1 and Parkin genes in mice and analyzing the NMJ innervation rate of the double knockout mice.

## Materials and methods

### Animals

Animal experiments were carried out in accordance with the animal care and use protocol approved by the Institutional Animal Care and Use Committee of University of Kansas Medical Center (KUMC) and in accordance with the Guidelines for the Care and Use of Laboratory Animals of KUMC. SOD1^G93A^ transgenic mice (JAX stock No. 004435, high copy number of transgene SOD1^G93A^), *pink1* knockout mice (JAX stock No. 017946), and *park2* knockout mice (JAX stock No. 006582) were purchased from the Jackson Laboratory (Bar Harbor, Maine, USA) and maintained in the animal facility at KUMC until analysis. Three to five animals were analyzed per genotype per age, and the animal numbers of animals are reported in the appropriate figure legend or the Results Section.

SOD1^G93A^ mice on a C57BL/6J background survive longer than those on an SJL/J background (Heiman-Patterson et al., 2005; Wooley et al., 2005). With the C57Bl/6J background, disease onset, as determined by the limb tremor, occurs between P91-111 (Dobrowolny et al., 2005; Hayworth and Gonzalez-Lima, 2009). The survival rate starts to fall below 100% around P125-130 (Heiman-Patterson et al., 2005; Wooley et al., 2005), and the mean survival duration is between P142-161 (Dobrowolny et al., 2005; Heiman-Patterson et al., 2005; Wooley et al., 2005; Hayworth and Gonzalez-Lima, 2009). We analyzed the SOD1^G93A^ mice at three stages: presymptomatic stage (P57) (Wooley et al., 2005; Hayworth and Gonzalez-Lima, 2009), a stage with denervation (P85) (Dobrowolny et al., 2005), and a symptomatic stage (P140) (Dobrowolny et al., 2005; Hayworth and Gonzalez-Lima, 2009).

### Transgene copy number analysis

All male SOD1^G93A^ mice produced at our institute by mating SOD1^G93A^ mice with C57BL/6J female mice were analyzed for human SOD1 transgene copy number variation using the TaqMan qPCR genotype method as described in the Jackson Laboratory website (protocol name: Sod TgN Copy Number). Mice were excluded from analyses when they had low transgene copy numbers that showed more than a half cycle difference in qPCR (Leitner et al., 2009).

### Electron microscopy

The methods employed for electron microscopy have been described previously (Nishimune et al., 2004; Chen et al., 2011). In brief, diaphragm muscles and spinal cord at cervical level four were fixed in 5% glutaraldehyde, 4% paraformaldehyde in phosphate-buffered saline (PBS, pH 7.1, Delbucco's PBS), washed, refixed in 1% OsO_4_, dehydrated, and embedded in resin. Ultrathin transverse sections were cut by skipping 30 μm minimum between sections for muscles and 100 μm minimum between sections for spinal cords to avoid analyzing the same NMJs or motor neurons. Sections were stained with lead citrate and uranyl acetate and systematically scanned using a transmission electron microscope. All profiles of NMJs and motor neuron cell bodies encountered in the micrographs were quantified. Motor neuron cell bodies were reconstructed by stitching multiple electron micrographs. The levels of electron micrographs in **Figures 3A–D** were adjusted to maximize the gray scale range of the images.

Autophagosomes were defined as double membrane structures that sequester a portion of the cytoplasm and/or organelles at various stages of degeneration from the cytoplasm, as described in the Guidelines for the Use and Interpretation of Assays for Monitoring Autophagy (3rd edition) (Klionsky et al., 2016). As described in these guidelines, autophagy can be both selective and nonselective for the autophagosome content. Mitophagosomes were defined as autophagosomes containing normal or degenerating mitochondria (Klionsky et al., 2016). Degenerated mitochondria were defined as double-membrane organelles with cristae, in which the cristae are not aligned regularly in more than 50% of the mitochondria profile. Mitochondria with large vacuole-like spaces within or between the cristae were recognized as degenerated mitochondria.

### Immunohistochemistry and image analysis

The following antibodies were used: ATP synthase β subunit (A21351, Molecular Probes), choline acetyltransferase (AB144P, Millipore), Bnip3 (3769, Cell Signaling Tech.), LC3 (NB100-2220SS, Novus), neurofilament (2H3, DSHB, or N4142, Sigma), p62/SQSTM1 (2C11, Abnova), Pink1 (Abcam, ab75487), Parkin (sc-32282, Santa Cruz), SV2 (DSHB), Synaptophysin (18-0130, Zymed), Alexa Fluor 488 and 568 conjugated secondary antibodies, DAPI (4′,6-diamidino-2-phenylindole, dihydrochloride), and Alexa Fluor 594-conjugated α-bungarotoxin (Molecular Probes).

Immunohistochemical analyses have been described previously (Nishimune et al., 2004; Chen et al., 2011). Briefly, mice were fixed by transcardiac perfusion with 2% paraformaldehyde in PBS. Muscles and T12-L1 portions of the lumbar spinal cord were removed and post-fixed in 2% paraformaldehyde at room temperature, washed with PBS, and cryoprotected in 20% sucrose/PBS before being frozen in Optimal Cutting Temperature compound (Sakura, Torrance, CA), and sections were cut using a cryostat (transverse for lumbar spinal cords and both transverse and longitudinal for muscles). Muscles and lumbar spinal cords were sectioned at a thickness of 20 μm, blocked in PBS containing 2% bovine serum albumin (BSA), 2% normal goat serum, and 0.1–0.3% Triton X-100. For the goat anti-choline acetyltransferase antibody, BSA and normal goat serum were replaced with donkey serum for the blocking solution. Sections were then incubated with primary antibodies for 1 day at room temperature or 3 days at 4°C, washed with PBS, and incubated in appropriate secondary antibodies for 2 h at room temperature. Muscle sections were also incubated with Alexa Fluor 594-conjugated α-bungarotoxin. Lumbar spinal cord sections were also incubated with DAPI. Sections were then washed with PBS and mounted using VectaShield (Vector, Burlingame, CA).

Whole-mount immunohistochemical staining of diaphragm muscles was performed as follows. Muscles were fixed in 2% paraformaldehyde/PBS, washed in PBS, permeabilized in 0.5% Triton/0.1 M glycine/PBS overnight at 4°C, and blocked for 48 h at 4°C in 2% BSA/2% normal goat serum/0.5% Triton/PBS. Tissues were incubated with primary antibodies (anti-neurofilament and anti-SV2) for 48 h at 4°C, washed with PBS, and incubated with Alexa Fluor 488-conjugated anti-mouse IgG1 secondary antibody along with Alexa Fluor 594-conjungated α-bungarotoxin for 24 h at 4°C. After final washing, tissues were mounted in mounting medium (glycerol, PBS, *p*-phenylenediamine).

#### NMJs with LC3 puncta

Immunohistochemical staining of LC3 showed puncta with strong signal intensity in presynaptic terminals of NMJ profiles. The NMJs were identified in transverse sections of diaphragm muscles by staining the nerve with anti-neurofilament and SV2 antibodies, and the acetylcholine receptors with Alexa Fluor 594-conjugated α-bungarotoxin. The number of NMJs with LC3 punctate signal overlapping with the nerve signal was quantified and described as the percentage of total NMJs counted.

#### Immunohistochemical signal intensity quantification

The signal intensities of autophagy- and mitophagy-related proteins in motor neurons were measured using the method described in our previous publication (Chen et al., 2011). In brief, confocal images were obtained using Nikon A1R confocal microscope using PlanApo 20x, NA = 0.75 or PlanFluor 40x, NA = 1.30 lenses. The circumference of the motor neuron cell bodies in the lumbar spinal cord transverse sections was defined by the anti-choline acetyltransferase staining patterns. The average signal intensity within motor neurons was measured using the Show Region Statistics function in MetaMorph software ver. 7.0 (Molecular Devices).

The signal intensities of ATP synthase β subunit in the presynaptic terminals of NMJs were measured using the method described above. The presynaptic terminals of NMJs in the muscle sections were defined by anti-neurofilament and anti-synaptophysin staining patterns. The average signal intensity within the presynaptic terminals of NMJs was measured using the Show Region Statistics function in MetaMorph software. The observer was blinded for the genotype.

#### P62 aggregation

Motor neuron cell bodies were identified using ChAT staining to identify their location in the ventral horn of the lumbar spinal cord. The number of motor neurons that displayed large p62 positive structures was quantified and described as a percentage of total motor neurons counted.

#### NMJs with axon swelling

Adjacent to NMJs, axons have a constant diameter similar to the axon diameter that branches from the axon fascicle (Court et al., 2008; Samuel et al., 2012). Axons with abnormal enlargement near NMJs that is greater than the axon near the fascicle were qualitatively assessed, and the number of NMJs with abnormally enlarged axons were quantified as NMJs with axon swelling.

#### Fragmented NMJs

We used the analytical criteria described previously by Valdez et al. (Valdez et al., 2010; Taetzsch et al., 2017). Briefly, fragmented acetylcholine receptors were defined as acetylcholine receptor cluster with five or more islands and/or a segment of receptor cluster showing small and/or irregularly shaped receptor clusters. In young adult NMJs, acetylcholine receptor clusters show pretzel-like distribution patterns that are connected and not separated.

#### Denervation analysis

The denervation analysis has been described previously (Chen et al., 2012). Briefly, muscle sections were stained using antibodies against nerves (anti-neurofilament and anti-SV2) and Alexa Fluor 594-conjugated α-bungarotoxin for acetylcholine receptors. Adult motor nerve terminals show perfect overlap with acetylcholine receptor clusters, which indicates fully innervated NMJs. NMJs were assessed for areas of the acetylcholine receptor clusters that were not occupied by nerves, whether in part or in full, as partially innervated NMJs or denervated NMJs. Quantifications are from four mice of each genotype and more than 98 NMJs per animal. The observer was blinded for the genotype.

### Statistics

All statistics were performed using GraphPad Prism software version 6. Significance was assessed by un-paired *t*-test or one-way ANOVA with Tukey's multiple comparison test. The *p-* and *n-*values are reported in the text. All data in the graphs are shown as the mean ± S.E.M.

## Results

### Increased autophagosomes at ALS NMJs

**Figure 1:**
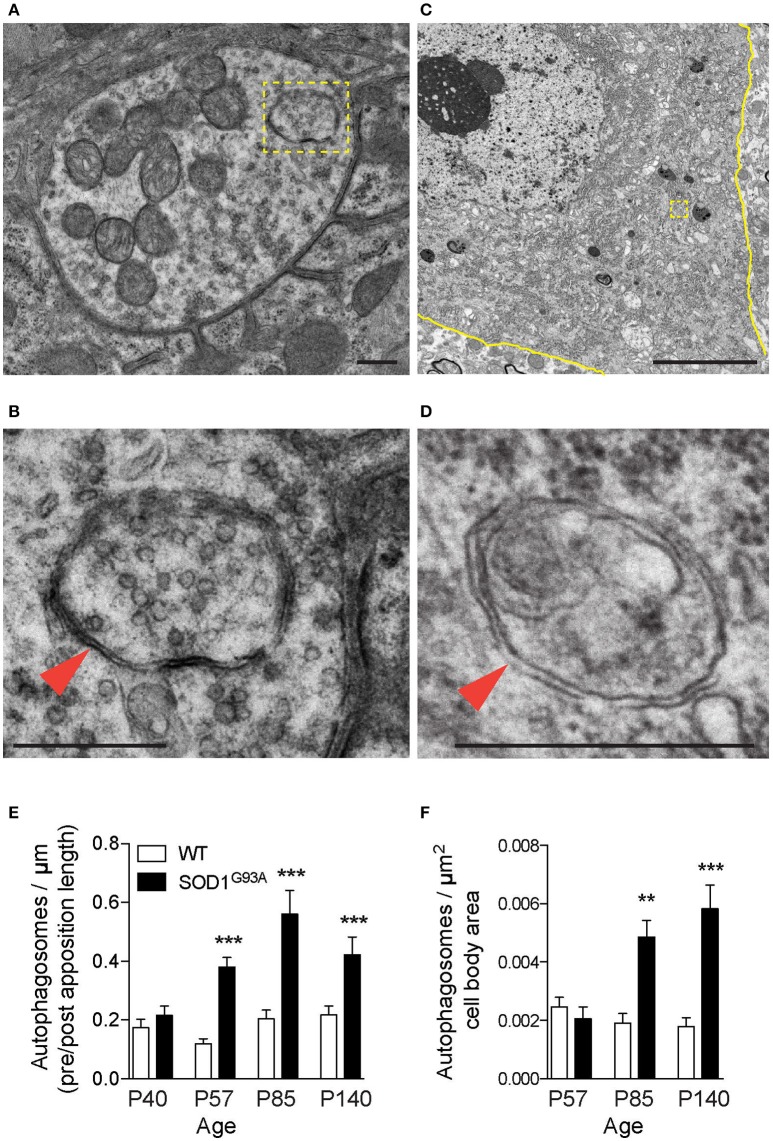
Autophagosomes increase at NMJs in SOD1^G93A^ mice. Representative electron micrographs of autophagosomes in SOD1^G93A^ mice in **(A,B)** the presynaptic terminals of NMJs at P57 and **(C,D)** motor neuron cell bodies at P140. Higher-magnification images of **(A,C)** are shown in **(B,D)**. The colored marks on the micrographs are as follows: plasma membrane of motor neuron cell bodies (yellow) and autophagosomes (yellow boxes and orange arrowheads). Scale bars: **(A,B,D)** = 500 nm, **(C)** = 5 μm. Quantification revealed significantly increased numbers of autophagosomes in **(E)** the presynaptic terminals of NMJs and **(F)** motor neuron cell bodies of SOD1^G93A^ mice compared to wild-type mice (WT). The number of autophagosomes was normalized by pre- and postsynaptic apposition length for NMJs (synapse profile size) or the area of motor neuron cell bodies (μm^2^). Graphs show mean ± SEM indicated by whiskers. Asterisks indicate significant differences by one-way ANOVA with Tukey's multiple comparison test (**p < 0.01, ***p < 0.001). NMJ quantifications are from *n* = five animals and 80–123 NMJs for each age and each genotype. Motor neuron cell body quantifications are from *n* = two animals for P57, three animals for P85, five animals for P140, and 40–73 cell bodies for each age and each genotype.

Autophagy-related genes are mutated in ALS patients, which suggested altered autophagy function in motor neurons. Therefore, autophagosomes in motor neurons of SOD1^G93A^ mice were analyzed using electron microscopy. The diaphragm was analyzed because ALS patients suffer from respiratory problems (Braun, 1987; Arnulf et al., 2000; Miller et al., 2009; Tateishi et al., 2010). NMJs in diaphragms of SOD1^G93A^ mice revealed double-membraned autophagosomes containing synaptic vesicles and mitochondria at the nerve terminals (Figures 1A,B, **3A**, Supplementary Figure 1). The number of autophagosomes in NMJ profiles were significantly higher in the presynaptic terminals of SOD1^G93A^ NMJs compared to those of age- and sex-matched wild-type mice beginning in the presymptomatic stage at postnatal day (P) 57, at stage with denervation (P85), and at symptomatic stage (P140) (Figure 1E; P40: SOD1^G93A^, 0.22 ± 0.031 autophagosome/synapse profile size (μm), WT, 0.17 ± 0.029; P57: SOD1^G93A^, 0.38 ± 0.033, WT, 0.12 ± 0.018; P85: SOD1^G93A^, 0.56 ± 0.080, WT, 0.20 ± 0.030; P140: SOD1^G93A^, 0.42 ± 0.061, WT, 0.22 ± 0.031; mean ± S.E.M.). Spinal cords of the same set of animals were dissected at cervical level four to analyze the motor neuron cell bodies of phrenic nerves innervating the diaphragms. Similar autophagosomes were detected in the cell bodies of motor neurons (Figures 1C,D). The number of autophagosomes in motor neuron cell bodies in SOD1^G93A^ mice were similar at P57 but significantly higher at P85 and P140 compared to those of age- and sex-matched wild-type mice (Figure 1F; P57: SOD1^G93A^, 0.0021 ± 0.00040 autophagosomes/μm^2^, WT, 0.0025 ± 0.00034; P85: SOD1^G93A^, 0.0049 ± 0.00057, WT, 0.0019 ± 0.00033; P140: SOD1^G93A^, 0.0058 ± 0.00081, WT, 0.0018 ± 0.00031). These results indicate that autophagosomes accumulate in the presynaptic terminals of NMJs earlier than in motor neuron cell bodies in SOD1^G93A^ mice.

### Increased levels of autophagosome marker at ALS NMJs

**Figure 2:**
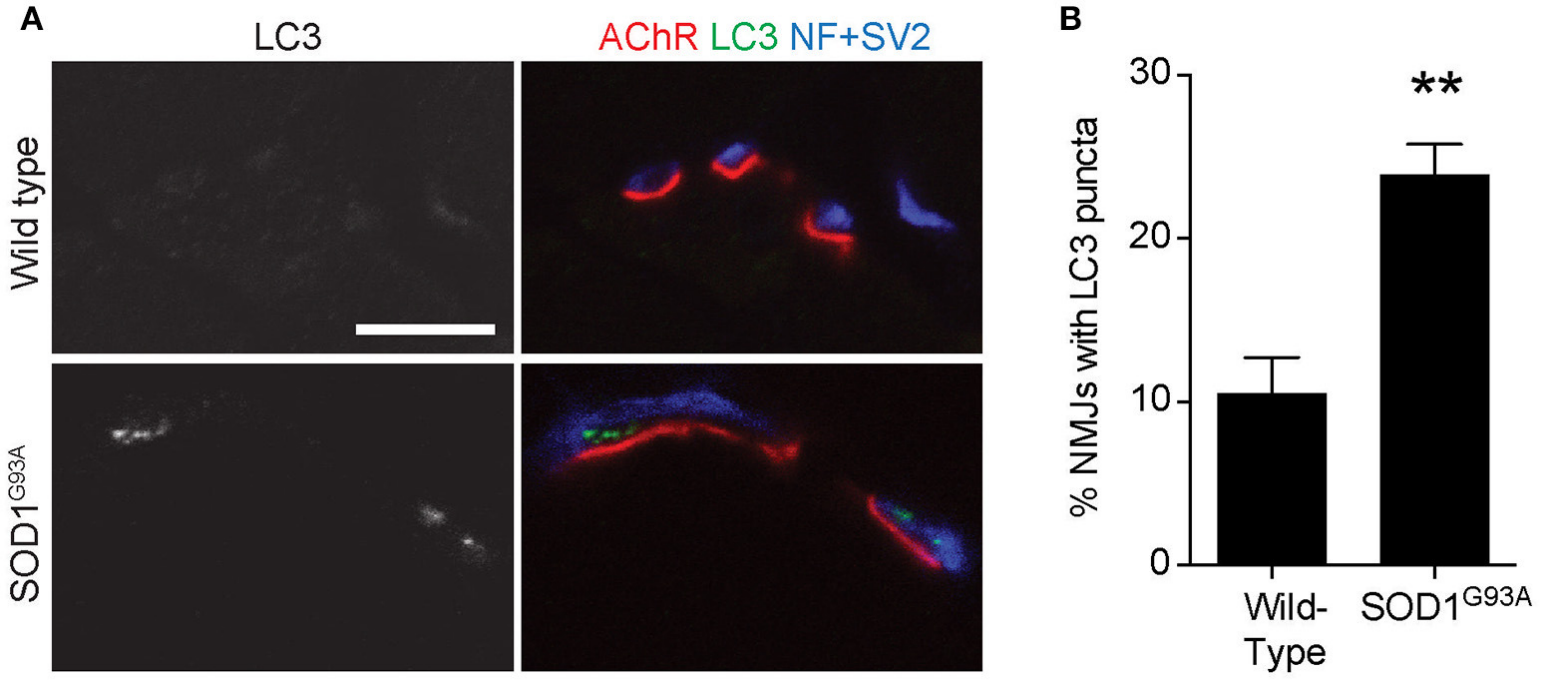
Accumulation of an autophagosome marker in the presynaptic terminals of NMJs of SOD1^G93A^ mice **(A)** Representative confocal micrographs of immunohistochemistry showing accumulation of the autophagosome marker LC3 (grayscale in left panels, green in right panels) in presynaptic terminals of diaphragm NMJs in SOD1^G93A^ mice at P120, but not at NMJs of wild-type mice at P140. Nerves were stained using anti-neurofilament and anti-SV2 antibodies (blue), and postsynaptic acetylcholine receptors were labeled with Alexa 594-conjugated α-bungarotoxin (red). In these cross-section images of NMJs, presynaptic terminals are above the bungarotoxin signal, and postsynaptic myotubes are below the bungarotoxin signal. Scale bar: 10 μm. **(B)** The number of NMJs with LC3 puncta was significantly higher in SOD1^G93A^ mice than in wild-type mice. Quantifications are from *n* = three animals and 268–305 NMJs each genotype in confocal images. Asterisks indicate a significant difference by un-paired *t*-test (**p < 0.01).

To validate the autophagosome accumulation in the presynaptic terminals of NMJs detected by electron microscopy, immunohistochemistry was used to detect an autophagosome marker, LC3 (microtubule-associated protein 1 light chain 3; Kabeya et al., 2000). LC3 proteins accumulated as large puncta in NMJ presynaptic terminals in diaphragms of SOD1^G93A^ mice (Figure 2A lower panels). However, these LC3 puncta were rarely detected in the presynaptic terminals of wild-type NMJs (Figure 2A upper panels). The proportion of NMJs bearing LC3 puncta in the presynaptic terminal was significantly higher in SOD1^G93A^ mice (23.9 ± 1.9%) than in wild-type mice (10.5 ± 2.2%, Figure 2B). These results suggest accumulation of autophagosomes in the NMJ presynaptic terminals of SOD1^G93A^ mice and are consistent with the electron microscopic quantifications.

### Mitochondria degeneration and mitophagy at ALS NMJs

**Figure 3:**
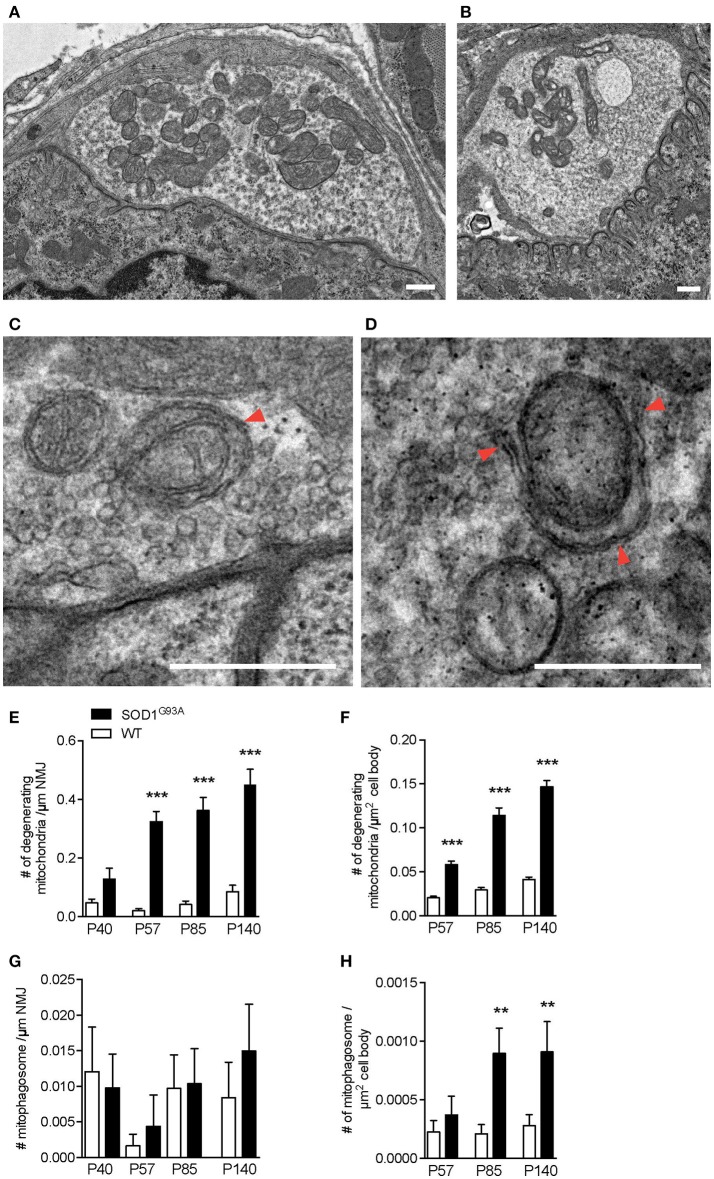
Numbers of degenerating mitochondria in the presynaptic terminals of NMJs and in motor neurons. Representative electron micrographs of **(A)** a NMJ in wild-type mouse, **(B)** degenerated mitochondria in a NMJ of a SOD1^G93A^ mouse, **(C)** mitophagosomes (arrowhead) near the presynaptic membrane in a NMJ of wild-type mouse, and **(D)** a phagophore (arrowheads) around degenerated mitochondria in a NMJ of a SOD1^G93A^ mouse. Scale bar: 500 nm. Quantification of electron micrographs revealed significantly increased numbers of degenerated mitochondria in **(E)** NMJs and **(F)** motor neuron cell bodies and of mitophagosomes in **(G)** NMJs (*p* = 0.6295) and **(H)** motor neuron cell bodies of SOD1^G93A^ mice (black bar) compared to age- and sex-matched wild-type mice (white bar). Graphs show mean ± SEM. Asterisks indicate significant differences (**p < 0.005, ***p < 0.0001) between SOD1^G93A^ mice and wild-type mice at each age by one-way ANOVA with Tukey's multiple comparison test. Numbers of animals, NMJs, and motor neuron cell bodies are the same as in Figure 1.

The early increase in autophagosomes in the presynaptic terminals of NMJs suggested that the initiation of NMJ degeneration occurred during the presymptomatic stage in SOD1^G93A^ mice. To further investigate the degeneration in ALS motor neurons, the electron micrographs of NMJs and motor neuron cell bodies used in Figure 1 were analyzed for mitochondria. NMJs in diaphragms of SOD1^G93A^ mice contained significantly higher number of degenerated mitochondria in the presynaptic terminals that were still innervating endplates. The degenerated mitochondria had irregular cristae spacing, large vacuoles or degenerated cristae inside a double membrane structure, which were not detected in normal mitochondria in the NMJ presynaptic terminals of age-and sex-matched wild-type mice (Figures 3A,B). The representative example SOD1^G93A^ NMJ contained an autophagosome in the presynaptic terminal, but the autophagosome did not contain any degenerated mitochondria. The numbers of degenerated mitochondria at NMJs were 3–15 times higher in SOD1^G93A^ mice than in that of wild-type mice at the presymptomatic stage P57 and remained higher at P85 and P140 [Figure 3E; P40: SOD1^G93A^, 0.13 ± 0.038 mitochondria/synapse profile size (μm), WT, 0.047 ± 0.013; P57: SOD1^G93A^, 0.32 ± 0.034, WT, 0.021 ± 0.0069; P85: SOD1^G93A^, 0.36 ± 0.045, WT, 0.042 ± 0.012; P140: SOD1^G93A^, 0.45 ± 0.052, WT, 0.085 ± 0.022]. In the same sets of animals, the numbers of degenerated mitochondria in the cell bodies of motor neurons were three to four times greater in SOD1^G93A^ mice than in wild-type mice among from P57 to P140 (Figure 3F; P57: SOD1^G93A^, 0.05814 ± 0.0041 degenerated mitochondria/μm^2^, WT, 0.020 ± 0.0017; P85: SOD1^G93A^, 0.11 ± 0.0087, WT, 0.029 ± 0.0028; P140: SOD1^G93A^, 0.15 ± 0.0069, WT, 0.041 ± 0.0026).

As we have shown previously, the number of autophagosomes increased significantly in ALS motor neurons and NMJs; however, the degenerated mitochondria do not seem to be degraded efficiently by mitophagy in SOD1^G93A^ mice. Mitophagy mediated degradation of damaged mitochondria has been shown to occur locally in distal axons (Ashrafi et al., 2014). Therefore, we searched for mitophagosomes in the electron micrographs and identified clear examples of mitophagy in the NMJ presynaptic terminals of wild-type mice and SOD1^G93A^ mice (Figures 3C,D). A representative micrograph of a wild-type NMJ shows synaptic vesicles accumulating near the presynaptic membrane and an active zone, and dark synaptic cleft and postsynaptic junctional folds are visible (Figure 3C). A mitophagosome was identified near the presynaptic membrane and was surrounded by synaptic vesicles (an arrowhead in Figure 3C). A representative micrograph of a SOD1^G93A^ NMJ shows a phagophore surrounding a degenerated mitochondrion with irregular cristae (arrowheads in Figure 3D). Degenerated mitochondria and synaptic vesicles are visible nearby. Therefore, the number of mitophagosome was analyzed in the presynaptic terminals of NMJs and motor neuron cell bodies. However, mitophagosome number in NMJ was not different between SOD1^G93A^ mice and age- and sex-matched wild-type mice among all the stages analyzed, between P40–140 (Figure 3G; P40: SOD1^G93A^, 0.0098 ± 0.0047 mitophagosome / synapse profile size (μm), WT, 0.012 ± 0.0062; P57: SOD1^G93A^, 0.0044 ± 0.0044, WT, 0.0016 ± 0.0016; P85: SOD1^G93A^, 0.010 ± 0.0049, WT, 0.0097 ± 0.0047; P140: SOD1^G93A^, 0.015 ± 0.0066, WT, 0.0084 ± 0.0050). These results suggest insufficient function of mitophagy in the presynaptic terminals of ALS NMJs despite the accumulation of degenerated mitochondria. Meanwhile, in motor neuron cell bodies, the number of mitophagosomes was higher in SOD1^G93A^ mice than in wild-type mice at P85 (Figure 3H; P57: SOD1^G93A^, 0.00037 ± 0.00016 mitophagosomes/μm^2^, WT, 0.00022 ± 0.000097; P85: SOD1^G93A^, 0.00090 ± 0.00022, WT, 0.00021 ± 0.000080; P140: SOD1^G93A^, 0.00091 ± 0.00026, WT, 0.00028 ± 0.000095). However, there were significantly fewer mitophagosomes than autophagosomes in motor neuron cell bodies of SOD1^G93A^ mice (compare Figures 1F, 3H). In addition, the numbers of mitophagosomes increased more slowly than the increase in degenerated mitochondria in motor neuron cell bodies (compare Figures 3F,H). Together, these results suggest decreased function of mitophagy in SOD1^G93A^ mice, especially at NMJs, and this may lead to the accumulation of degenerated mitochondria in the presynaptic terminals of NMJs.

### Reduction in mitophagy-related proteins in ALS motor neurons

**Figure 4:**
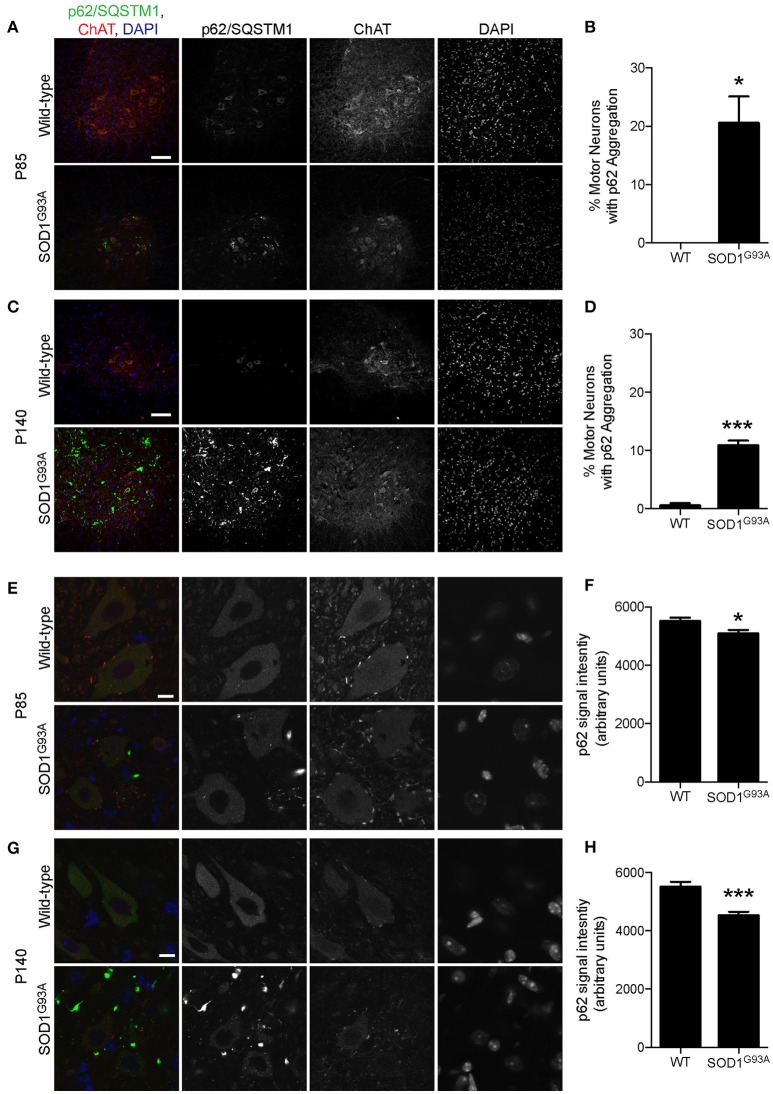
Aggregation of p62/SQSTM1 in a subpopulation of motor neurons despite lower p62 levels in SOD1^G93A^ mice. **(A–D)** Aggregation of p62/SQSTM1 was observed in a greater percentage of motor neuron cell bodes of the lumbar spinal cord of SOD1^G93A^ mice compared with age- and sex-matched wild-type controls. Representative confocal micrographs of immunohistochemistry for p62/SQSTM1, choline acetyltransferase (ChAT), and DAPI are shown for **(A)** P85 and **(C)** P140 mice. Quantification of the percentage of motor neuron cell bodies with p62/SQSTM1 aggregates is shown for **(B)** P85 and **(C)** P140 mice and includes 46–86 motor neurons at P85 and 45–101 motor neurons at P140 from *n* = three animals for each genotype. **(E–H)** Decreased intensity of p62/SQSTM1 was observed in motor neuron cell bodes of the lumbar spinal cord of SOD1^G93A^ mice compared with age- and sex-matched wild-type controls. Representative confocal micrographs of immunohistochemistry for p62/SQSTM1, choline acetyltransferase (ChAT), and DAPI are shown for **(E)** P85 and **(G)** P140 mice. Quantification of average signal intensity shows decreased intensity of p62/SQSTM1 in motor neuron cell bodies for **(F)** P85 and for **(H)** P140 mice. Quantification for signal intensity are from *n* = three animals include 47–56 motor neurons for P85 **(F)** and 45–50 motor neurons for P140 **(H)** mice for each genotype. Graphs show mean ± SEM. Asterisks indicate significant differences as analyzed by un-paired *t*-test (*p < 0.05, ***p < 0.001) **(B,D,F,H)**. Different confocal scanning conditions were used between the two ages; therefore, the signal intensity arbitrary units should not be compared between ages. Scale bar: 100 μm **(A,C)** and 10 μm **(E,G)**.

p62/Sequestosome 1 (SQSTM1) is mutated in ALS patients (Fecto et al., 2011; Hirano et al., 2013; Teyssou et al., 2013). p62/SQSTM1 has a ubiquitin binding domain at the C-terminus and accumulates to depolarized and ubiquitinated mitochondria (Geisler et al., 2010; Okatsu et al., 2010). p62/SQSTM1 recruits LC3 to mitochondria to induce mitophagy (Geisler et al., 2010). Pink1/Parkin-mediated mitophagy is dependent on p62/SQSTM1 and VDAC1 (Geisler et al., 2010). Interestingly, a large number of p62 aggregated structure was observed among motor neurons and non-motor neuron cells in the gray matter of lumbar spinal cord of SOD1^G93A^ mice at P85, which was hardly detected in wild-type age- and sex-matched control mice (Figure 4A). This feature was more prevalent in SOD1^G93A^ mice at P140 compared to that of P85 (compare lower panels in Figures 4A,C).

First, we examined the number of motor neuron cell bodies of the lumbar spinal cord that displayed large p62/SQSTM1 aggregated structures. At P85, 20.5 ± 4.6% of motor neuron cell bodies of SOD1^G93A^ mice contained p62/SQSTM1 aggregated structures compared to zero observed in wild-type control mice (Figures 4A,B). At P140, 10.4 ± 0.8% of motor neuron cell bodies in SOD1^G93A^ mice displayed large p62/SQSTM1 aggregated structures compared to 0.5 ± 0.5% of that in wild-type control mice (Figures 4C,D).

Next, we used immunohistochemistry to quantify the expression level of p62/SQSTM1 specifically in motor neuron cell bodies without the contamination from other cells in the gray matter. Importantly, the expression level of p62/SQSTM1 in motor neuron cell bodies estimated by average signal intensity was 10.3% lower at P85 and 19.7% lower at P140 in the lumbar spinal cord of SOD1^G93A^ mice compared to that of wild-type control mice (Figures 4E–H, P85: SOD1^G93A^, 4,936 ± 130; WT, 5,501 ± 133; P140: SOD1^G93A^, 4,415 ± 119; WT, 5,496 ± 185; arbitrary unit). The decreased protein level of p62/SQSTM1 suggested a reduction of autophagy and/or mitophagy function in SOD1^G93A^ motor neurons.

**Figure 5:**
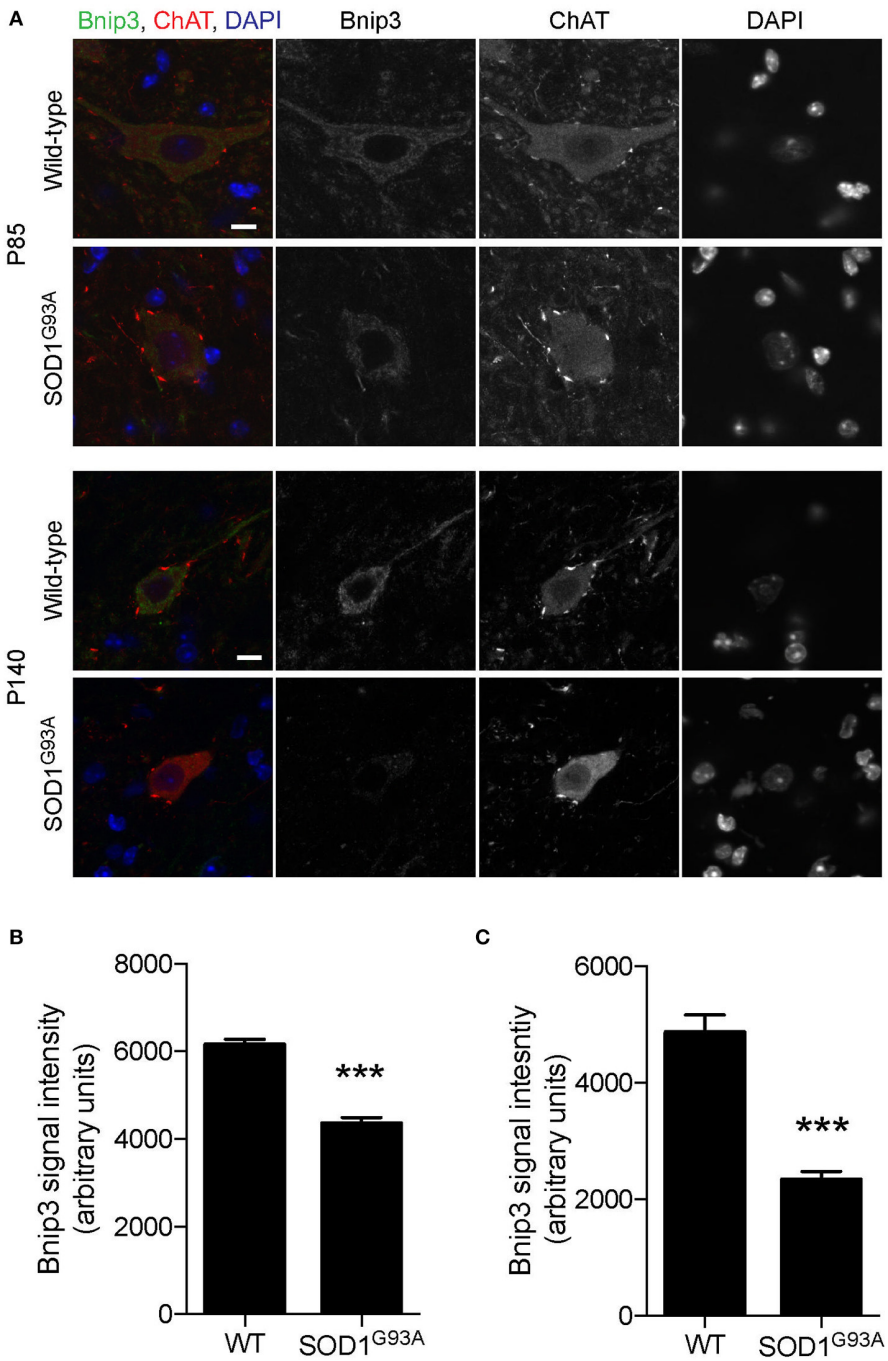
Decreased expression of Bnip3 in motor neurons in SOD1^G93A^ mice. Bnip3 protein expression levels were significantly reduced in motor neurons in lumbar spinal cords of SOD1^G93A^ mice compared to age- and sex-matched wild-type controls. Representative confocal micrographs of immunohistochemistry for Bnip3, choline acetyltransferase (ChAT), and DAPI are shown for P85 and P140 mice **(A)**. Quantification for signal intensity is shown for **(B)** P85 and **(C)** P140 mice. Quantification for signal intensity are from *n* = three animals include 42–56 motor neurons for P85 and 31–34 motor neurons for P140 mice for each genotype. Graphs show mean ± SEM. Asterisks indicate significant differences as analyzed by un-paired *t*-test (***p < 0.001). Different confocal scanning conditions were used between the two ages; therefore, the signal intensity arbitrary units should not be compared between ages. Scale bar: 10 μm.

To investigate whether mitophagy function is altered in motor neurons of SOD1^G93A^ mice, expression levels of mitophagy-related proteins were analyzed using immunohistochemistry to specifically detect protein levels in motor neurons. Bnip3 (BCL2 and adenovirus E1B 19-kDa-interacting protein 3) forms a homodimer and is integrated into the mitochondrial outer membrane by C-terminal transmembrane domains. The LC3-interacting region in the N-terminal domain interacts with LC3-II at the phagophores to induce mitophagy (Springer and MacLeod, 2016). The expression level of Bnip3 in motor neuron cell bodies was 29% lower at P85 and 52% lower at P140 in lumbar spinal cords in SOD1^G93A^ mice compared to that of age- and sex-matched wild-type control mice at P85 and P140 (Figure 5; P85: SOD1^G93A^, 4,343 ± 140; WT, 6,151 ± 124; P140: SOD1^G93A^, 2,335 ± 144; WT, 4,864 ± 301; arbitrary unit).

**Figure 6:**
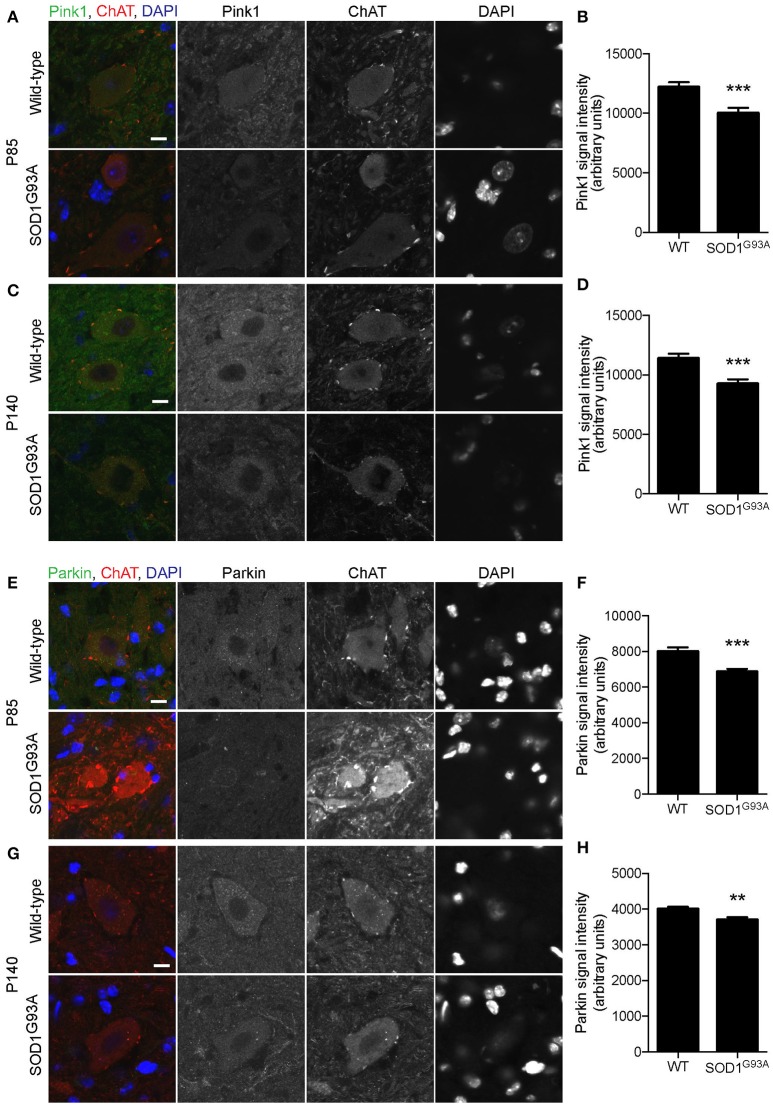
Decreased expression of mitophagy-related proteins in motor neurons of SOD1^G93A^ mice. Pink1 and Parkin protein expression levels were significantly reduced in motor neurons in lumbar spinal cords of SOD1^G93A^ mice compared to age- and sex-matched wild-type controls. **(A–D)** Representative confocal micrographs of immunohistochemistry for Pink1, choline acetyltransferase (ChAT), and DAPI are shown for **(A)** P85 and **(C)** P140 mice. Quantification for signal intensity is shown for Pink1 at P85 **(B)** and P140 **(D)** mice. Quantification for signal intensity are from *n* = three animals include 37–43 motor neurons for P85 and 41–50 motor neurons for P140 mice for each genotype. **(E–H)** Representative confocal micrographs of immunohistochemistry for Parkin, choline acetyltransferase (ChAT), and DAPI are shown for **(E)** P85 and **(G)** P140 mice. Quantification for signal intensity is shown for Parkin at P85 **(F)** and P140 **(H)** mice. Quantification for signal intensity are from *n* = three animals include 32–38 motor neurons for P85 and 28–43 motor neurons for P140 for each genotype. Graphs show mean ± SEM. Asterisks indicate significant differences as analyzed by un-paired *t*-test (**p < 0.01, ***p < 0.001) **(B,D,F,H)**. Different confocal scanning conditions were used between the two ages; therefore, the signal intensity arbitrary units should not be compared between ages. Scale bar: 10 μm.

Mitochondrial protein PTEN-induced putative kinase 1 (Pink1) is degraded rapidly in normal mitochondria, but is stabilized at the surface of depolarized mitochondria, where it recruits and activates Parkin (Matsuda et al., 2010; Narendra et al., 2010). Activated Parkin ubiquitinates mitochondrial outer membrane proteins, which recruit LC3 to initiate mitophagy (Geisler et al., 2010). Pink1 levels were significantly lower in motor neuron cell bodies of lumbar spinal cord of SOD1^G93A^ mice at P85 and P140 (18 and 19% lower, respectively) compared to that of age- and sex-matched wild-type control mice (Figures 6A–D; P85: SOD1^G93A^, 10,013 ± 447; WT, 12,199 ± 407; P140: SOD1^G93A^, 9,241 ± 390; WT, 11,385± 395; arbitrary unit). Furthermore, Parkin signal intensity in motor neuron cell bodies was significantly lower in motor neuron cell bodies of lumbar spinal cords of SOD1^G93A^ mice at P85 and P140 (14 and 7% lower, respectively) compared to wild-type control mice (Figures 6E–H; P85: SOD1^G93A^, 6,851 ± 171; WT, 7,988 ± 239; P140: SOD1^G93A^, 3,698 ± 76; WT, 4,004 ± 61; arbitrary unit). These protein-level analyses also suggested a reduction of mitophagy function in motor neurons of SOD1^G93A^ mice.

### Pink1/parkin double knockout mice exhibit severe degeneration of NMJs in young adults

The potential reduction of mitophagy function may lead to accumulation of degenerated mitochondria at NMJs and dying-back neuropathy of ALS motor neurons in SOD1^G93A^ mice. We have identified that the levels of p62/SQSTM1, Bnip3, Pink1, and Parkin are significantly reduced in motor neuron cell bodies of SOD1^G93A^ mice (Figures 4–6). p62/SQSTM1 is necessary for Pink1/Parkin-mediated mitophagy (Geisler et al., 2010). Therefore, double knockout mice (DKO) mice for Pink1 (*pink1*) and Parkin (*park2*) were analyzed to investigate the role of mitophagy in NMJ maintenance.

**Figure 7:**
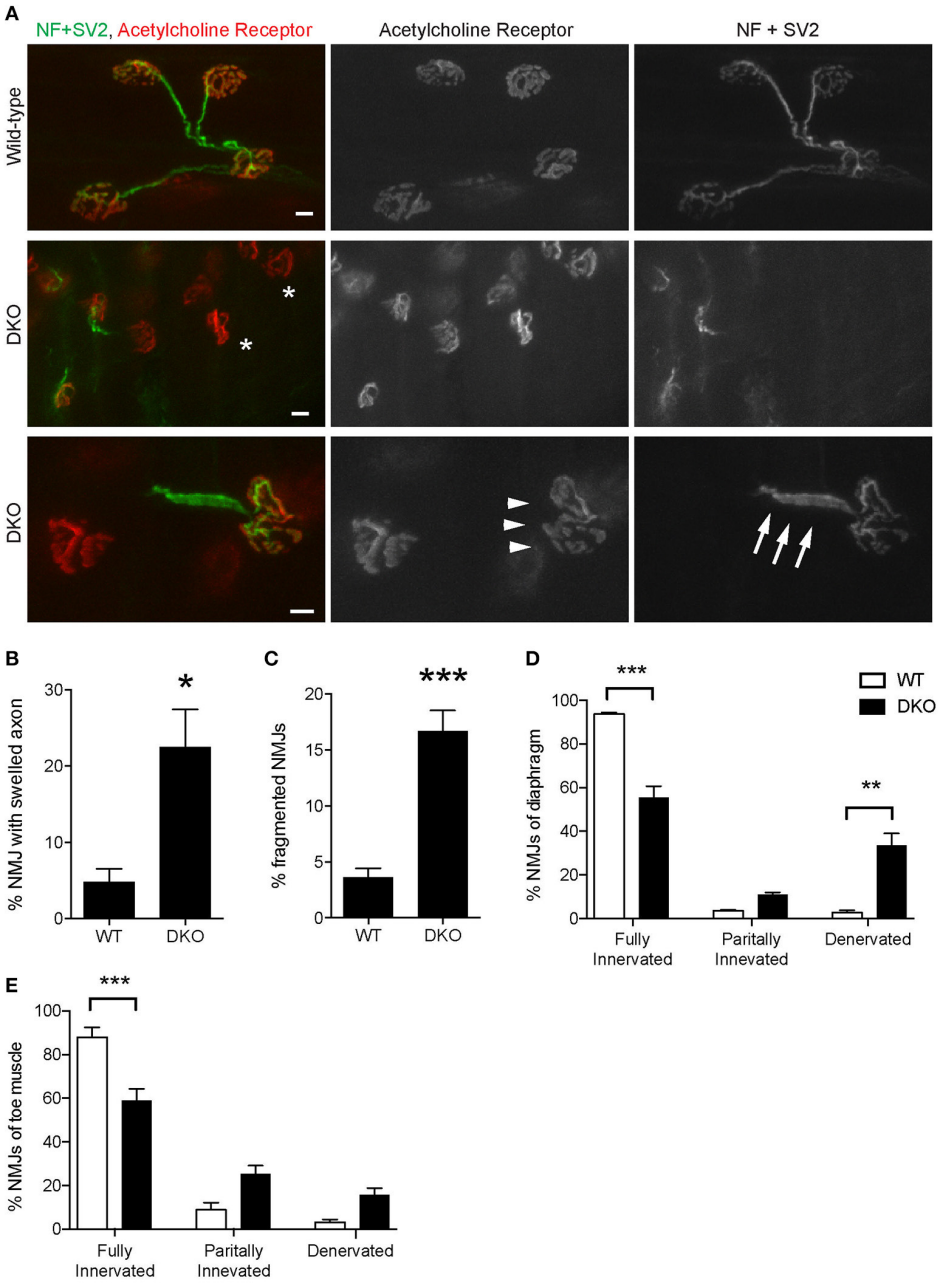
Pink1/Parkin DKO mice have denervated NMJs. **(A)** Representative confocal images of individual NMJs in diaphragms of age-matched wild-type control mouse (top row) and Pink1/Parkin DKO mouse (middle and bottom rows). DKO diaphragm showed significantly increased NMJ denervation (white asterisks). NMJs of DKO mice showed axon swelling (white arrows) and fragmentation of acetylcholine receptor cluster (white arrowheads) at ages between P85 and P110. NMJs of wild-type mice of the same age did not show these phenotypes. Quantifications of **(B)** NMJ with axon swelling and **(C)** fragmented NMJs are from *n* = four animals (3 male, 1 female) for each genotype, and **(B)** 45–161 NMJs and **(C)** 58–206 NMJs were counted for each animal. NMJ denervation rate of DKO mice was compared to wild-type mice in diaphragm **(D)** and toe **(E)** muscles. Quantification data are presented as a percent of NMJs that are either fully innervated, partially innervated, or denervated. Quantifications are from *n* = four animals for each genotype and **(D)** 175–407 NMJs and **(E)** 98–212 NMJs were counted for each animal. Graphs show mean ± SEM. Asterisks indicate significant differences as analyzed by un-paired *t*-test **(C,D)** and one-way ANOVA with Tukey's multiple comparison test **(D,E)** (*p < 0.05, **p < 0.01, ***p < 0.001). Scale bar: 10 μm.

The loss of Pink1 and Parkin caused significant degeneration of NMJs in young adult mice. Diaphragm NMJs were analyzed at ages between P85 and P110 using whole-mount immunohistochemistry to minimize potential damage to NMJs due to sectioning. We have not detected premature death of DKO mice for Pink1 and Parkin to the age of 3.5 months. Both presynaptic nerve terminals and postsynaptic specialization showed abnormalities. Axons adjacent to NMJs showed swelling at a significantly higher rate in the DKO mice (22.5 ± 4.5%) than in age matched wild-type mice (4.9 ± 1.7%, Figures 7A,B). Postsynaptic acetylcholine receptors are clustered in a pretzel-like morphology that are mostly connected to each other in the muscles of wild-type mice. However, NMJs in the DKO mice showed fragmentation of acetylcholine receptor clusters at a significantly higher rate (16.7 ± 1.8%) than in wild-type NMJs (4.9 ± 1.7%, Figures 7A,C). Importantly, the DKO mice had significantly lower numbers of fully innervated NMJs in diaphragm (55.6 ± 5.1%) than wild-type mice (93.7 ± 0.8%, Figure 7D). Denervated NMJs were significantly more common in the DKO mice (33.5 ± 5.5%) but were rarely detected in wild-type mice (2.8 ± 1.0%). Furthermore, the toe muscle of DKO mice showed similar NMJ innervation rate and had significantly lower numbers of fully innervated NMJs (58.9 ± 5.5%) than wild-type mice (88.0 ± 4.5%, Figure 7E). The increased NMJ denervation is less likely to be caused by muscle fiber degeneration and regeneration because the muscle fiber cross-sectional area was not significantly different between the DKO mice and wild-type control mice (DKO, 7,146 ± 309 μm^2^; WT 6,225 ± 369 μm^2^), and central nuclei was not observed in muscle fibers. These data demonstrate that mitophagy is essential for the maintenance of NMJ innervation.

**Figure 8:**
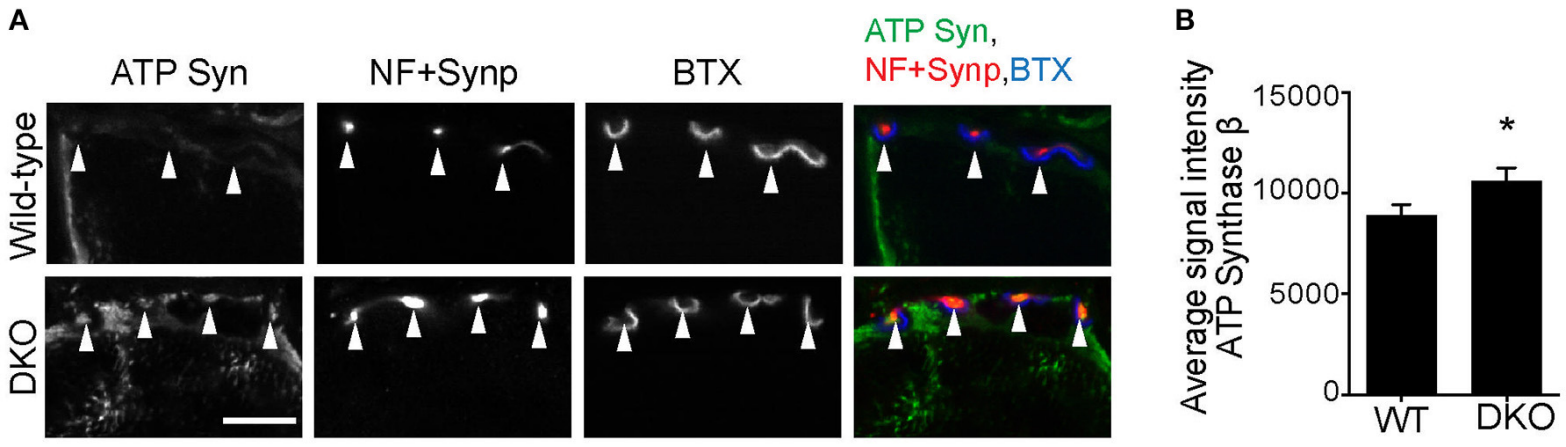
Higher level of ATP synthase protein in presynaptic terminals of Pink1/Parkin DKO mice. **(A)** Representative confocal micrographs of immunohistochemistry for mitochondrial protein ATP synthase β subunit (ATP Syn) in presynaptic terminals (arrowheads) of tibialis anterior NMJs in the Pink1/Parkin DKO mice at ages between P85 and P110 compared to wild–type mice of the same age. Presynaptic terminals of NMJs were defined by anti-neurofilament and anti-synaptophysin staining pattern (NF + Synp). Postsynaptic acetylcholine receptors were labeled with Alexa 594-conjugated α-bungarotoxin (BTX). In these cross-section images of NMJs, presynaptic terminals are above the bungarotoxin signal, and postsynaptic myotubes are below the bungarotoxin signal. Scale bar: 10 μm. **(B)** Average signal intensity of ATP synthase β subunit is greater in presynaptic terminals of Pink1/Parkin DKO mice than in those of wild-type mice. Quantifications are from *n* = four animals and 97–101 NMJs for each genotype in confocal images. Graph shows mean ± SEM. Asterisks indicate significant differences as analyzed by un-paired *t*-test (*p < 0.05).

Finally, we asked whether reduced mitophagy cause accumulation of mitochondria at motor nerve terminals. Immunohistochemical signal intensity of the ATP synthase β subunit was measured at NMJ presynaptic terminals. The ATP synthase signal intensity was significantly higher in the DKO mice than in wild-type mice (DKO, 729.3 ± 10.86; WT, 499.8 ± 12.54; arbitrary unit, Figure 8). These results suggest that a lack of one of the mitophagy pathways, here, Pink1 and Parkin, results in the accumulation of mitochondria at nerve terminals. The reduced mitophagy function and the accumulation of degenerated mitochondria is likely to cause NMJ denervation in ALS model SOD1^G93A^ mice.

## Discussion

Ultrastructural analysis revealed clear examples of mitophagy in the presynaptic terminals of NMJs of wild-type mice and SOD1^G93A^ mice (Figure 3). To our knowledge, this report is the first to show electron micrographs of mitophagosomes at mammalian NMJs. We identified significant increases in the number of autophagosomes and degenerated mitochondria in the NMJ presynaptic terminals of SOD1^G93A^ mice compared to wild-type controls. The accumulation of degenerated mitochondria at NMJs is consistent with that observed at ALS patient NMJs (Siklos et al., 1996). However, mitophagosome number did not increase significantly, and the expression levels of mitophagy-related proteins, p62/SQSTM1, Bnip3, Pink1, and Parkin were significantly lower in SOD1^G93A^ mice than in aged- and sex-matched wild-type controls. These results suggest a reduced function of mitophagy in the NMJ presynaptic terminals of SOD1^G93A^ mice, which may lead to accumulation of degenerated mitochondria, NMJ denervation, and dying back neuropathy. Consistent with this hypothesis, Pink1 and Parkin DKO mice exhibited NMJ denervation at young adult ages and increased immunohistochemical signal intensity of the ATP synthase β subunit, which suggested an accumulation of mitochondria in the NMJ presynaptic terminals. To our knowledge, this study is the first report to test the effect of deleting both Pink1 and Parkin in the maintenance of NMJs. Overexpression of human wild-type SOD1 in mice has been reported to cause mitochondrial vacuolization and axonopathy around 1 year of age (Jaarsma et al., 2000). We have not analyzed the phenotype of the transgenic mouse overexpressing wild-type SOD1 to compare against that of SOD1^G93A^ mice, which is a limitation of the current study.

We have detected both autophagosomes and mitophagosomes in the presynaptic terminals of wild-type NMJs by using electron microscopy. This observation is consistent with a previous report that describes mitophagy in the distal axons of hippocampal neurons (Ashrafi et al., 2014). These functions of autophagy and mitophagy at the distal end of axons allow local mechanisms to preserve the function of nerve terminals without relying on the mechanisms in the cell body, which is a long distance away. However, the mitophagy function seems to show defects in presynaptic terminals of ALS NMJs and is not capable of depleting the accumulation of degenerated mitochondria. This reduced mitophagy function may be related to the lysosomal deficits that have been identified in SOD1^G93A^ mice (Xie et al., 2015).

As a potential mechanism of the mitophagy defect, we revealed the down regulation of p62 in motor neurons of SOD1^G93A^ mice. However, a previous publication reported progressive accumulation of p62 in lumbar spinal cord of SOD1^G93A^ mice at P125 (Gal et al., 2007). We also observed increased level of p62 aggregates in the lumbar spinal cords of SOD1^G93A^ mice at P85 and P140, but the increased signal derived mostly from cells other than motor neurons (Figure 4). The discrepancy between the two studies seems to stem from the difference in the antibodies used to detect p62/SQSTM1 protein. The specificity of the anti-p62/SQSTM1 antibody used in our study was verified using knockout mouse tissue in our previously published study (Yang et al., 2016). The antibody used by Gal et al. (Santa Cruz, SQSTM1 antibody, sc-10117) has not been verified using knockout animals and recognizes a second non-specific band in the western blot analysis, based on the information on the manufacturer's website. This antibody has been discontinued; therefore, the two antibodies could not be compared at this time.

As dysregulation autophagy has been shown in spinal cords of ALS patients, autophagy modulation has been explored as a potential treatment for ALS motor neuron dysfunction. Results of studies stimulating autophagy have been somewhat conflicting. Enhancing autophagy using rapamycin to inhibit mammalian target of rapamycin (mTOR) was shown to worsen ALS related symptomology, decrease life span, and result in a more severe mitochondrial dysfunction, possibly activating apoptosis, in SOD1^G93A^ mice (Zhang et al., 2011). While another study showed that rapamycin treatment of SOD1^G93A^ mice had no effect on survival, but did increase survival of SOD1^G93A^ mice devoid of mature lymphocytes, indicating a delicate interplay with the immune system (Staats et al., 2013). Rapamycin also worsens ALS symptomology in valosin-containing protein mutant mice (Ching and Weihl, 2013), and had no effect on SOD1 H46R/H48Q mutant mice (Bhattacharya et al., 2012), both separate mouse models of ALS. However, enhancing autophagy using trehalose, independent of mTOR, delayed disease onset in SOD1^G93A^ mice, increased life span, reduced motor neuron loss in spinal cord, reduced NMJ denervation, and preserved mitochondrial function (Castillo et al., 2013; Zhang et al., 2014; Li et al., 2015). Furthermore, TAR DNA-binding protein 43 (TDP43) mutations have been identified in familial ALS patients, and animal and cell culture models have been studied. Enhancing autophagy increased cell survival *in vitro* of primary rat neurons transfected with TDP43(A315T)-GFP or human iPSC-derived motor neurons with TDP43 M337V mutation (Barmada et al., 2014). In addition, enhancing autophagy in mice overexpressing wild-type TDP43 alleviated the progression motor function loss (Wang et al., 2012). Taken together these results provide promise that modulating autophagy may be a treatment option for ALS.

This study has shown that the reduced mitophagy function and the accumulation of degenerated mitochondria are likely to contribute to the dying-back neuropathy of ALS NMJs. Further analyses are necessary to elucidate the mechanism by which mitophagy is down regulated in ALS motor neurons. Homeostatic feedback loop signals link the biogenesis of mitochondria and the level of mitophagy (Palikaras et al., 2015). Therefore, better understanding of the mitophagy defect can aid in the development of a novel intervention to ameliorate mitochondrial defects in ALS.

## Author contributions

RR, WD, HN designed the work; RR, ST, TT, YB, LN, HW, HMN, WD, HN acquired, analyzed, and interpreted the data; RR, HN wrote the manuscript. All participated in the final approval of the manuscript.

### Conflict of interest statement

The authors declare that the research was conducted in the absence of any commercial or financial relationships that could be construed as a potential conflict of interest.

## Supplementary material

Supplementary Figure 1 Examples of autophagosomes at NMJs in SOD1^G93A^ mice. Representative electron micrographs of autophagosomes in SOD1^G93A^ mice in the presynaptic terminals of NMJs at P57. Orange arrowheads point to the autophagosomes. **(D,E,F)** Higher-magnification images of autophagosomes in **(A,B,C)**. **(A,D)** Double membrane autophagosome structure surrounds synaptic vesicles between the presynaptic membrane and mitochondria, which contains cristae. **(B,E)** In the four membrane autophagosome structure, the outside double membrane has not closed yet. An autophagosome forms a double-membrane structure or a structure with multiple layers of double-membrane (Klionsky et al., 2016). Given the large evidence suggesting that autophagosomes are originated from endoplasmic reticulum membrane, these structures may reflect the early formation stage of autophagosomes. **(C,F)** Four membrane autophagosome structure surrounds synaptic vesicles between the presynaptic membrane and degenerating mitochondria. Scale bars: 500 nm.
