# Supplementary figures and images for: Impaired Mitophagy Plays a Role in Denervation of Neuromuscular Junctions in ALS Mice
Source: Front Neurosci. 2017 Aug 25;11:473. doi: 10.3389/fnins.2017.00473 (PMC5575151; doi:10.3389/fnins.2017.00473)

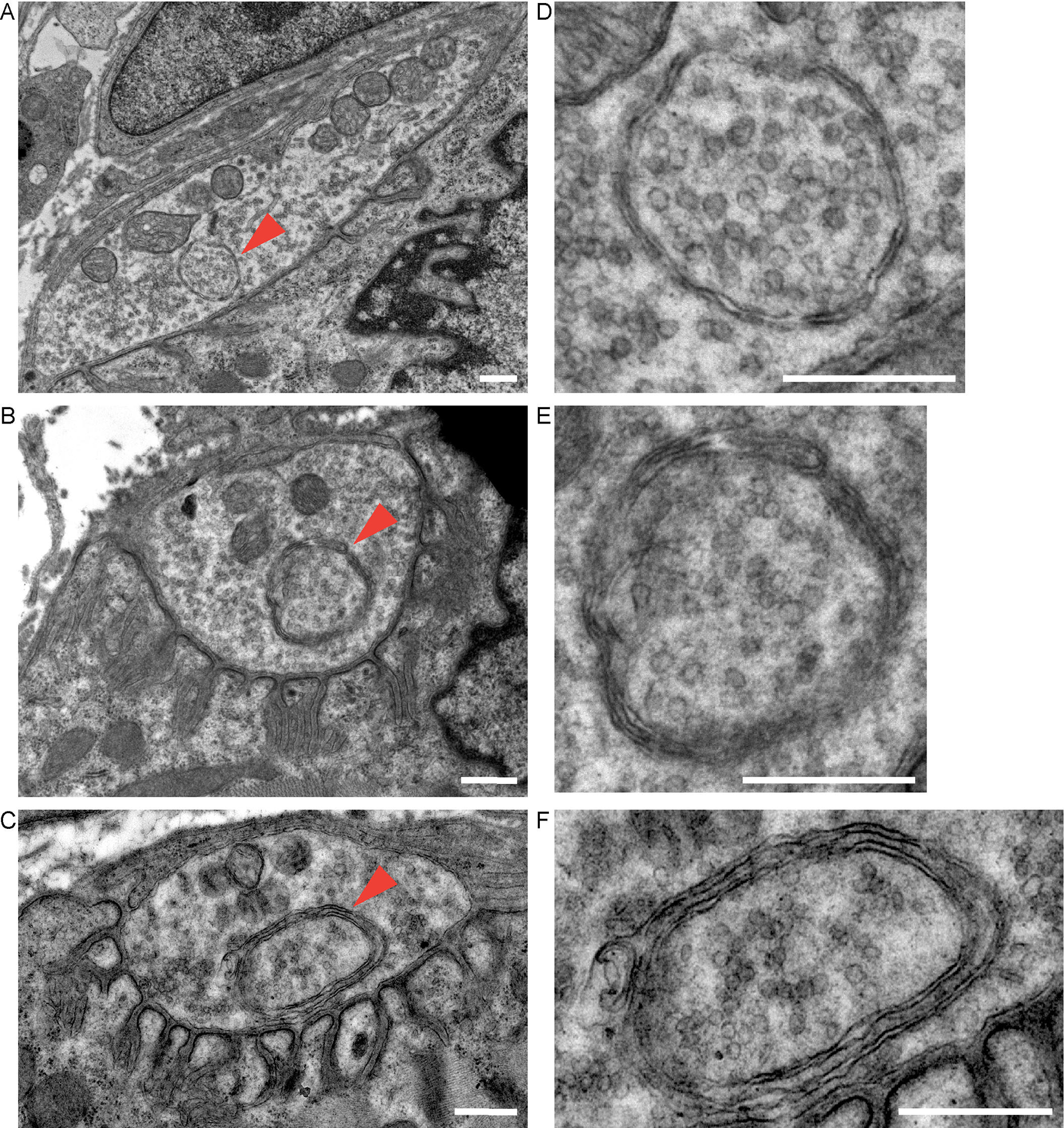

Supplement: Supplementary Figure 1 — Examples of autophagosomes at NMJs in SOD1G93A mice. Representative electron micrographs of autophagosomes in SOD1G93A mice in the presynaptic terminals of NMJs at P57. Orange arrowheads point to the autophagosomes. (D,E,F) Higher-magnification images of autophagosomes in (A,B,C). (A,D) Double membrane autophagosome structure surrounds synaptic vesicles between the presynaptic membrane and mitochondria, which contains cristae. (B,E) In the four membrane autophagosome structure, the outside double membrane has not closed yet. An autophagosome forms a double-membrane structure or a structure with multiple layers of double-membrane (Klionsky et al., 2016). Given the large evidence suggesting that autophagosomes are originated from endoplasmic reticulum membrane, these structures may reflect the early formation stage of autophagosomes. (C,F) Four membrane autophagosome structure surrounds synaptic vesicles between the presynaptic membrane and degenerating mitochondria. Scale bars: 500 nm. [file Image1.TIF]
